# Hybrid-Mechanism Synergistic Flexible Nb_2_O_5_@WS_2_@C Carbon Nanofiber Anode for Superior Sodium Storage

**DOI:** 10.3390/nano14070631

**Published:** 2024-04-05

**Authors:** Yang Zhao, Ziwen Feng, Yipeng Tan, Qinglin Deng, Lingmin Yao

**Affiliations:** 1 School of Physics and Materials Science, Guangzhou University, Guangzhou 510006, China; 2112119105@e.gzhu.edu.cn (Y.Z.); 2112119037@e.gzhu.edu.cn (Z.F.); 2112119039@e.gzhu.edu.cn (Y.T.); 2 Research Center for Advanced Information Materials (CAIM), Huangpu Research & Graduate School of Guangzhou University, Guangzhou 510555, China; 3 Joint Institute of Guangzhou University & Institute of Corrosion Science and Technology, Guangzhou University, Guangzhou 510275, China

**Keywords:** Nb_2_O_5_, WS_2_, free-standing, synergistic effects, sodium-ion batteries, anode

## Abstract

Sodium-ion batteries (SIBs) have demonstrated remarkable development potential and commercial prospects. However, in the current state of research, the development of high-energy-density, long-cycle-life, high-rate-performance anode materials for SIBs remains a huge challenge. Free-standing flexible electrodes, owing to their ability to achieve higher energy density without the need for current collectors, binders, and conductive additives, have garnered significant attention across various fields. In this work, we designed and fabricated a free-standing three-dimensional flexible Nb_2_O_5_@WS_2_@C carbon nanofiber (CNF) anode based on a hybrid adsorption–intercalation–conversion mechanism of sodium storage, using electrospinning and hydrothermal synthesis processes. The hybrid structure, aided by synergistic effects, releases the advantages of all materials, demonstrating a superior rate performance (288, 248, 211, 158, 90, and 48 mA h g^−1^ at the current density of 0.2, 0.5, 1, 2, 5, and 10 A g^−1^, respectively) and good cycling stability (160 mA h g^−1^ after 200 cycles at 1 A g^−1^). This work provides certain guiding significance for future research on hybrid and flexible anodes of SIBs.

## 1. Introduction

Due to the growing fossil energy crisis and environmental pollution problems, green and non-polluting renewable energy sources are developing rapidly [1,2]. In particular, rechargeable batteries have been widely used in areas such as removable electronic devices and electric vehicles [3,4]. However, lithium-ion batteries (LIBs), which are the most widely used rechargeable batteries, are commercially limited due to lower crustal abundance and higher prices [5,6]. Sodium-ion batteries (SIBs) are currently considered as a potential alternative to LIBs due to their low cost and their similar electrochemical mechanism to LIBs [7]. However, due to the larger radius and slow diffusion kinetics of sodium ions compared to lithium ions, commercially available graphite anodes cannot be directly used for SIBs, and there is an urgent need to develop new high-performance anodes for SIBs [8,9,10].

Currently, the research on anodes for SIBs can be mainly classified into three main categories, namely alloy-based, conversion-based, and intercalation-based, according to the different mechanisms of sodium storage [11,12]. Among them, alloy-based anode materials, including Sn and Sb, undergo an alloying reaction during sodium storage and have high theoretical specific capacity and low operating voltage. However, the substantial volume changes during the alloying–dealloying reaction of sodium ions can significantly impact the sodium storage performance of these materials [13,14]. Conversion-based anode materials undergo a phase transition during charging and discharging, and the conversion reaction is a multi-electron-transfer process, so they have a high specific capacity. Common materials include CuO and MoS_2_. However, they also face some drawbacks, such as poor rate performance and large volume expansion [15,16,17]. Intercalation-based anode materials are based on the intercalation mechanism, and most of them have lower theoretical specific capacity, but they show excellent rate performance and cycle stability performance with less volume expansion during the charging and discharging process. Typical anode materials for intercalation-based SIBs include titanium-based oxides (e.g., TiO_2_) and niobium-based oxides (e.g., Nb_2_O_5_) [18,19]. All three types of sodium storage materials have their advantages and disadvantages. Current research focuses on how to highlight the advantages and exclude the disadvantages of each material, which is the main direction of SIB anode material research.

In this study, we designed and prepared a hybrid SIBs anode material, denoted as Nb_2_O_5_@WS_2_@C CNFs. This electrode material contains four different components: hard carbon, Nb_2_O_5_, WS_2_, and soft carbon. Among them, hard carbon has become the most widely used anode electrode material for SIBs because of its wide availability and low price [20]. Its sodium-ion storage mechanism based on adsorption (sloping region, above 0.1 V vs. Na^+^/Na)–intercalation (plateau region, below 0.1 V vs. Na^+^/Na) gives it a stable structure and cycling stability but also limits its specific capacity [18,20,21]. Orthorhombic Nb_2_O_5_ is a typical layered material with intercalation behavior, featuring abundant intercalation sites internally; it can be utilized for the storage of lithium ions and sodium ions [22,23]. Recent studies have indicated that it serves as an outstanding energy storage anode with high rate capability and long cycling performance [24,25]. Transition metal dichalcogenides (MoS_2_, WS_2_, etc.) have extremely high theoretical capacities due to their distinct layer structure similar to graphite and conversion-based sodium storage mechanism [26,27]. However, the large volume expansion leads to a drastic capacity decay [16,28,29,30]. The incorporation of soft carbon significantly improves the conductivity and mitigates the volume expansion [31]. An electrode coupled with hybrid-mechanism synergistic effects has been certified in our previous work to achieve excellent sodium storage performances [18]. On this basis, this work compounded Nb_2_O_5_, WS_2_, CNFs, and soft carbon to prepare a Nb_2_O_5_@WS_2_@C CNF hybrid-mechanism anode. Ultimately, the free-standing flexible electrodes have exhibited excellent rate performance and cycling stability.

## 2. Materials and Methods

### 2.1. Materials

Niobium ethoxide (Nb(C_2_H_5_O)_5_, 99.95% trace metals basis), polyacrylonitrile (PAN, average Mw = 150,000), ammonium tetrathiotungstate ((NH_4_)_2_WS_4_, 99.9%), anhydrous glucose (99.5%), and N, N-dimethylformamide (DMF, 99%) were purchased from commercial sources and used without any further purification.

### 2.2. Synthesis of Nb_2_O_5_ CNFs

To obtain the precursor solution, 0.9 g Nb(C_2_H_5_O)_5_ and 0.75 g PAN were dissolved in 10 mL DMF by vigorous magnetic stirring for 12 h. Then, the precursor solution was poured into a plastic syringe, followed by electrospinning. The electrospinning experiments were carried out at a flow rate of 1.1 mL h^−1^ under a high voltage of 15 kV. Subsequently, the obtained precursor Nb_2_O_5_ carbon nanofibers were dried for 24 h at 70 °C and then preoxidized in air at 2 °C min^−1^ to 250 °C for 3 h. After that, the Nb_2_O_5_ CNFs were obtained at 2 °C min^−1^ to 700 °C for 5 h under an argon atmosphere in tube furnace. For comparison, samples prepared without adding Nb(C_2_H_5_O)_5_ were named pure CNFs.

### 2.3. Synthesis of Nb_2_O_5_@WS_2_ CNFs

Nb_2_O_5_@WS_2_ CNFs were synthesized by a typical solvothermal method. First, 25 mg of as-prepared Nb_2_O_5_ CNFs and 0.15 g of (NH_4_)_2_WS_4_ were added into 60 mL DMF. Subsequently, the mixture solution was put into a Teflon-lined stainless steel autoclave at 220 °C for 20 h. After repeated washing with deionized (DI) water and anhydrous ethanol, the sample was dried at 70 °C for 12 h. Finally, the Nb_2_O_5_@WS_2_ CNFs were gained at 2 °C min^−1^ to 650 °C for 5 h under an argon atmosphere.

### 2.4. Synthesis of Nb_2_O_5_@WS_2_@C CNFs

Anhydrous glucose was used as a carbon source to coat the previously prepared material by hydrothermal method. Typically, 20 mg of Nb_2_O_5_@WS_2_ CNFs and 20 mg of anhydrous glucose were added into 50 mL DI water and then heated to 180 °C for 12 h. Subsequently, the sample was dried at 70 °C for 12 h after repeated washing. Finally, Nb_2_O_5_@WS_2_@C CNFs were prepared at 2 °C min^−1^ to 650 °C for 5 h under an argon atmosphere.

### 2.5. Characterizations

The crystal structures of all samples were characterized through XRD (Rigaku, Tokyo, Japan). Raman spectra were acquired using a Raman spectrometer (Renishaw inVia, Dundee, IL, USA), while thermogravimetric analysis was conducted on a thermogravimetric analysis system (Netzsch TG 209 F3, Yokohama, Japan). The mass of C, O, and S elements was measured using an organic elemental analyzer (Elementar Vario EL cube, Langenselbold, Germany). SEM images and TEM images were captured using a scanning electron microscope (SEM, TESCAN MIRA LMS, Brno, Czech Republic) and a transmission electron microscope (TEM, FEI Talos F200X, Lausanne, Switzerland), respectively.

### 2.6. Electrochemical Tests

All samples were directly prepared as anodes for CR-2032 half-cells without the use of current collectors, binders, or conductive additives. Metal sodium discs were employed as both the working and reference electrodes, while a glass fiber membrane served as the separator. The electrolyte consisted of a 1 mol L^−1^ NaClO_4_ solution in propylene carbonate with 5% fluoroethylene carbonate. Electrochemical charge–discharge measurements were conducted using a Neware BTS-4000 battery (Shenzhen, China) testing system. CV profiles (with various scan rates ranging from 0.3 to 3 mV s^−1^) and EIS (with an amplitude of 5 mV and a frequency range from 10^5^ to 0.01 Hz) were obtained on a Solartron electrochemical workstation (Farnborough, UK).

## 3. Results and Discussion

**Figure 1:**
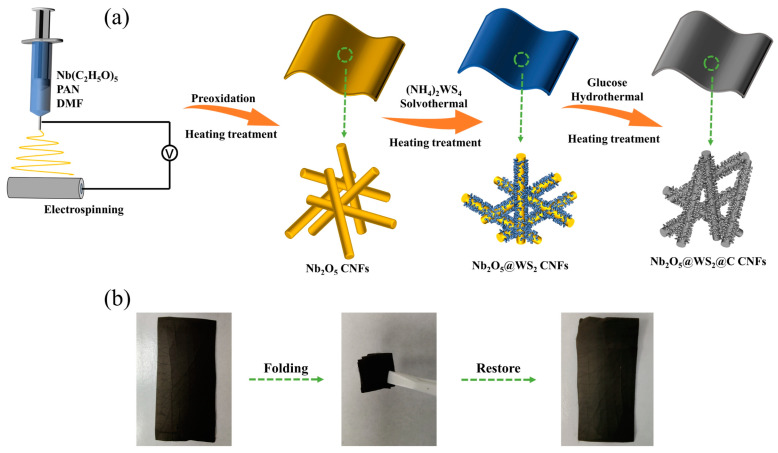
(**a**) Schematic illustration of the Nb_2_O_5_@WS_2_@C CNF film electrodes. (**b**) Photograph of Nb_2_O_5_@WS_2_@C CNF films which were folded many times.

The synthesis process of Nb_2_O_5_@WS_2_@C CNFs is schematically illustrated in Figure 1a. Initially, Nb_2_O_5_ CNFs were synthesized by the electrospinning and heating treatment method using polyacrylonitrile (PAN) and niobium ethoxide as the precursor. Subsequently, uniform growth of WS_2_ nanoflowers was achieved on the surface of Nb_2_O_5_ CNFs through the hydrothermal method followed by thermal processing. Finally, through hydrothermal processing followed by annealing, glucose-derived soft carbon was successfully coated onto the surface of the sample, resulting in the preparation of Nb_2_O_5_@WS_2_@C CNFs. Consistent with previous research [18], it was found that uniformly distributed Nb_2_O_5_ nanoparticles can be used as a buffer substance and can significantly enhance the flexibility of carbon nanofiber paper. As shown in Figure 1b and Figure S1, after being folded many times, the Nb_2_O_5_-based CNFs can regain their original appearance, while the pure CNFs fail, indicating that the addition of Nb_2_O_5_ nanoparticles has significantly improved the flexibility of the electrode.

**Figure 2:**
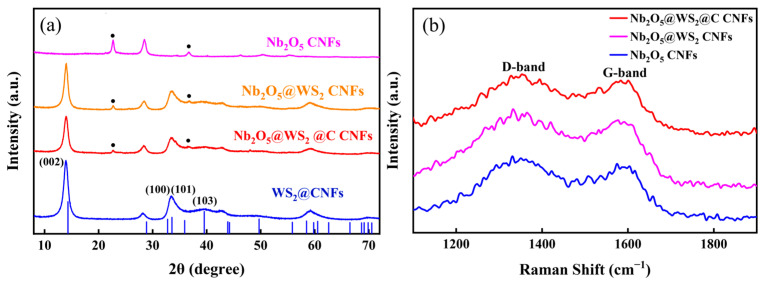
(**a**) XRD patterns of as-prepared samples (the peaks at the black dots indicate incorporation of Orthorhombic Nb_2_O_5_). (**b**) Raman shift of Nb_2_O_5_ CNFs, Nb_2_O_5_@WS_2_ CNFs, and Nb_2_O_5_@WS_2_@C CNFs.

Initially, the characteristic crystal face peaks on the X-ray diffraction (XRD) spectrum were utilized to confirm the successful preparation of the subsequent material and verify its crystal structure (Figure 2a). As shown in Figure 2a, all diffraction peaks of Nb_2_O_5_ CNFs can be well attributed to the orthorhombic phase of Nb_2_O_5_ (JCPDS No. 30-0873). CNFs@WS_2_ exhibits characteristic peaks located at 2θ = 14.1°, 33.5°, and 39.6° that can be assigned as the (002), (101), and (103) planes of 2H-WS_2_ (JCPDS No. 87-2417), respectively. In contrast, Nb_2_O_5_@WS_2_ CNFs with the addition of Nb_2_O_5_ not only exhibit a distinct 2H-WS_2_ peak but also display peaks corresponding to the orthorhombic Nb_2_O_5_ phase. This effectively confirms the successful preparation of Nb_2_O_5_@WS_2_ composite structured carbon fibers. Although Nb_2_O_5_@WS_2_@C CNFs show a slight decrease in peak intensity due to the presence of the coated carbon layer, there is no significant overall change. In order to investigate structural differences, we conducted a Raman spectroscopy analysis on the samples (Figure 2b). The two peaks located at approximately 1353 and 1590 cm^−1^ correspond to the D-band and G-band of carbon, respectively. By measuring the peak intensity ratio I_D_/I_G_, we can obtain information about the structural characteristics of the carbon material. The I_D_/I_G_ of Nb_2_O_5_ CNFs is approximately 1.25, while the I_D_/I_G_ values for Nb_2_O_5_@WS_2_ CNFs and Nb_2_O_5_@WS_2_@C CNFs are 1.28. This is attributed to the introduction of sulfur atoms, which slightly increases the material’s structural defects and enhances its degree of disorder, facilitating the diffusion of ions [32].

**Figure 3:**
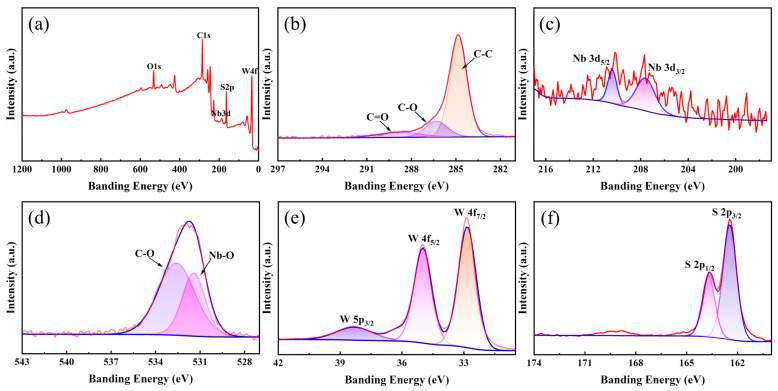
XPS spectra of the Nb_2_O_5_@WS_2_@C CNFs: (**a**) survey spectrum, (**b**) C 1s, (**c**) Nb 3d, (**d**) O 1s, (**e**) W 4f, and (**f**) S 2p.

To comprehend the phase composition and valence state of elements, X-ray photoelectron spectroscopy (XPS) tests were conducted on Nb_2_O_5_@WS_2_@C CNFs. Figure 3 illustrates typical XPS spectra. The comprehensive XPS survey clearly reveals the presence of C, Nb, O, W, and S elements (Figure 3a). Three peaks in the high-resolution spectrum of C 1s are located at about 284.8, 286.3, and 288.5 eV (Figure 3b), which derive from C-C, C-O, and C=O bonding, respectively. Meanwhile, the C-C peak was also used for the calibration of the entire spectrum. Figure 3c presents the deconvoluted spectra of Nb 3d orbitals, with two electronic signals located at 207.7 and 210.5 eV, attributed to Nb 3d_3/2_ and Nb 3d_5/2_ modes, respectively [33]. In the O1s spectrum, the fitted peaks located at 531.4 and 532.6 eV correspond to Nb-O and C-O bonds, respectively (Figure 3d). Then, in Figure 3e, the W 4f_7/2_ and W4f_5/2_ at 32.8 and 35 eV dominate the overall W signal, confirming the presence of W^4+^ in Nb_2_O_5_@WS_2_@C CNFs. The lower peak at 38.3 eV corresponds to W 5p_3/2_, indicating the presence of a small amount of W^6+^, which may be attributed to incompletely reacted WO_x_ [29,34,35]. In addition, the S 2p spectrum (Figure 3f) exhibits characteristic peaks at 162.5 and 163.7 eV, corresponding to the S 2p_3/2_ and S 2p_1/2_ states, respectively. Clearly, characterization results from XRD and XPS confirm the successful preparation of the envisioned Nb_2_O_5_@WS_2_@C CNFs.

**Figure 4:**
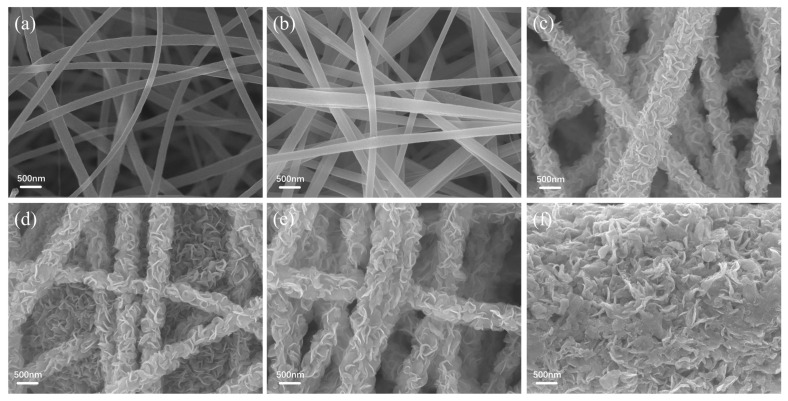
SEM images of (**a**) pure CNFs, (**b**) Nb_2_O_5_ CNFs, (**c**) Nb_2_O_5_@WS_2_ CNFs, (**d**) Nb_2_O_5_@WS_2_@C CNFs, (**e**) CNFs@WS_2_, and (**f**) pure WS_2_.

The microstructure and morphology of the samples were assessed through scanning electron microscopy (SEM) testing. As observed in Figure 4a,b, numerous fibers with a diameter of approximately 200 nm interweave with each other, forming a continuous mesh structure. In comparison to Nb_2_O_5_ CNFs and pure CNFs, the introduction of WS_2_ did not alter the network structure formed by interwoven fibers (Figure 4c). Compared to pure WS_2_ (Figure 4f), WS_2_ nanosheets are uniformly anchored on the surface of CNFs, forming a distinctive floral fiber structure. Evidently, this structure plays a significant role in improving the inhibition of WS_2_ aggregation. After the carbon coating of Nb_2_O_5_@WS_2_ CNFs, there is no apparent change in the overall microstructure of the material (Figure 4d).

**Figure 5:**
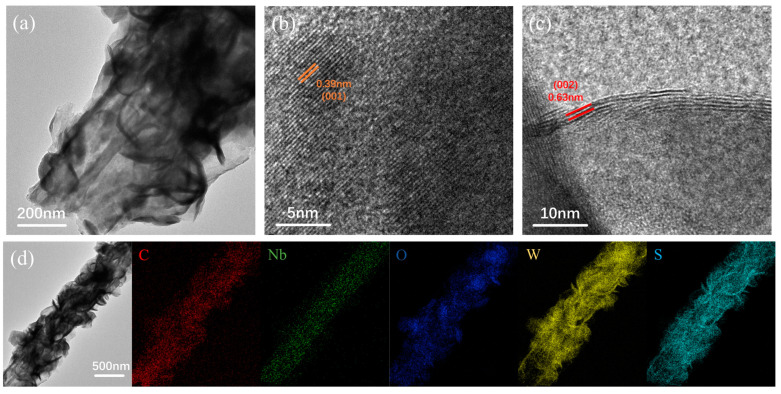
(**a**–**c**) TEM images of Nb_2_O_5_@WS_2_@C CNFs. (**d**) TEM image of Nb_2_O_5_@WS_2_@C CNFs with elemental mapping of C, Nb, O, W, and S.

To further investigate the crystal structure and elemental distribution of the material, we conducted transmission electron microscopy (TEM) and energy dispersive X-ray spectroscopy (EDS) testing on Nb_2_O_5_@WS_2_@C CNFs. As depicted in Figure 5a, a distinct composite structure of CNF, Nb_2_O_5_, WS_2_, and C layers is revealed, consistent with the observations from SEM. By conducting high-resolution TEM (HRTEM) imaging on different regions, as shown in Figure 5b, it was found that the 0.39 nm lattice spacing within the CNFs corresponds well to the (001) crystal plane of Nb_2_O_5_. Meanwhile, as shown in Figure 5c, the evident 0.63 nm lattice stripes are associated with 2H-WS_2_. Additionally, EDS mapping scans were conducted using transmission electron microscopy, and the results, as shown in Figure 5d, reveal a uniform distribution of Nb within the nanofibers, while W and S are predominantly concentrated on the nanosheets.

We attempted a quantitative analysis of the Nb_2_O_5_@WS_2_@C CNFs. Initially, we investigated the carbon content of PAN, which was found to be approximately 50 wt%. Subsequently, we calculated and attempted to configure Nb_2_O_5_/C in a mass ratio of 1:1 for the preparation of Nb_2_O_5_ CNFs. The validation was conducted through thermogravimetric analysis (TGA) (Figure S2). On this basis, a quantitative analysis of the elements C, O, and S in Nb_2_O_5_@WS_2_@C CNFs was conducted using an organic elemental analyzer. The calculated mass ratios for the CNF, Nb_2_O_5_, WS_2_, and C layers were found to be 11%, 11%, 72%, and 6%, respectively.

**Figure 6:**
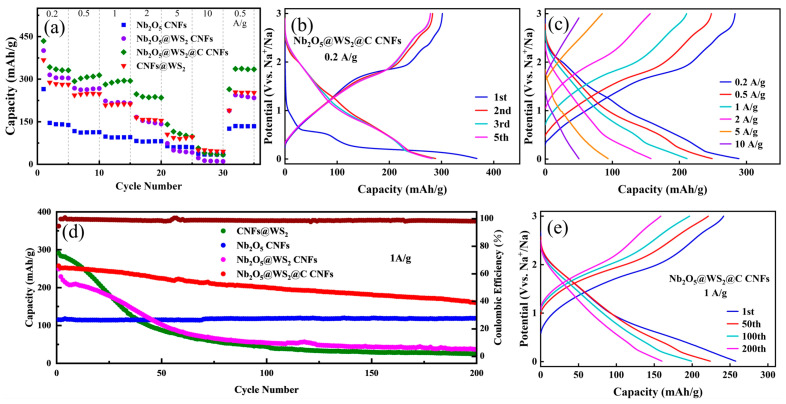
(**a**) Rate performance of as-prepared samples at different current densities. The GCD curve of (**b**) 1st, 2nd, 3rd, and 5th cycles at the current density of 0.2 A g^−1^ and (**c**) different current density for the Nb_2_O_5_@WS_2_@C CNFs. (**d**) Cycling stability of as-prepared samples at a current density of 1 A g^−1^ with (**e**) the GCD curve of the 1st, 50th, 100th, and 200th cycles.

The above-prepared flexible carbon nanofibers paper was used to fabricate CR-2032 dual-electrode half-cells for electrochemical performance testing. Initially, rate performance tests were conducted on cells with different anodes at various current densities ranging from 0.2 to 10 A g^−1^ (Figure 6a). In the initial galvanostatic charge–discharge (GCD) cycles, all anode electrodes exhibit higher specific capacity and lower Coulombic efficiency compared to subsequent cycles. This is attributed to the formation of the solid electrolyte interface (SEI) film and the irreversible insertion of sodium ions. Compared to Nb_2_O_5_ CNFs, with the assistance of WS_2_, Nb_2_O_5_@WS_2_ CNFs exhibit an increase in specific capacity from 141 mA h g^−1^ to 305 mA h g^−1^ at a current density of 0.2 A g^−1^. Additionally, the incorporation of glucose-derived carbon results in a slight decrease in specific capacity for Nb_2_O_5_@WS_2_@C CNFs at low current density. However, as the current density gradually increases, Nb_2_O_5_@WS_2_@C CNFs exhibit a higher specific capacity. Evidently, the addition of the carbon layer enhances the rate performance of the anode electrode. CNFs@WS_2_ exhibits excellent capacity density at low current density; however, the poor rate performance of WS_2_ results in its specific capacity being lower than that of the Nb_2_O_5_@WS_2_@C CNF electrode when the current density exceeds 5 A g^−1^. Figure 6c illustrates the GCD curves of the Nb_2_O_5_@WS_2_@C CNF electrode at current densities of 0.2, 0.5, 1, 2, 5, and 10 A g^−1^, demonstrating impressive specific capacities of 289, 249, 211, 158, 94, and 50 mA h g^−1^, respectively. Moreover, after the Nb_2_O_5_@WS_2_@C CNF electrode undergoes high current density charge–discharge cycles, its capacity can recover to 253 mA h g^−1^ when the current density returns to 0.5 A g^−1^, demonstrating outstanding reversibility in sodium-ion storage.

To investigate the cyclic performance of the electrodes, we subjected different electrodes to repeated charge–discharge cycles at the same current density. Figure 6d illustrates the cyclic charge–discharge performance of four electrodes at a current density of 1 A g^−1^ after activation. Clearly, despite CNFs@ WS_2_ having a higher initial specific capacity (291 mA h g^−1^), there is rapid capacity degradation during the cyclic process due to volume expansion and structural damage. After 200 cycles, only 23 mA h g^−1^ remains, resulting in a mere 8% capacity retention. In comparison, Nb_2_O_5_ CNFs demonstrate excellent cyclic stability and capacity retention. After combining the two and a carbon layer coating, the Nb_2_O_5_@WS_2_@C CNF electrode combines the advantages of different materials, exhibiting an initial high specific capacity of up to 257 mA h g^−1^. Even after 200 cycles, it still maintains a capacity of 160 mA h g^−1^. However, even though the Nb_2_O_5_@WS_2_@C CNFs exhibit much better cycling stability than CNFs@WS_2_ and Nb_2_O_5_@WS_2_ CNFs, the electrochemical reactions occurring within them are not entirely reversible. This is particularly evident from the GCD curves of the 1st, 50th, 100th, and 200th cycles, where although the Coulombic efficiency of each charge–discharge cycle exceeds 99%, irreversible capacity decay is still observed. This is primarily attributed to electrode material degradation and irreversible sodium-ion insertion.

**Figure 7:**
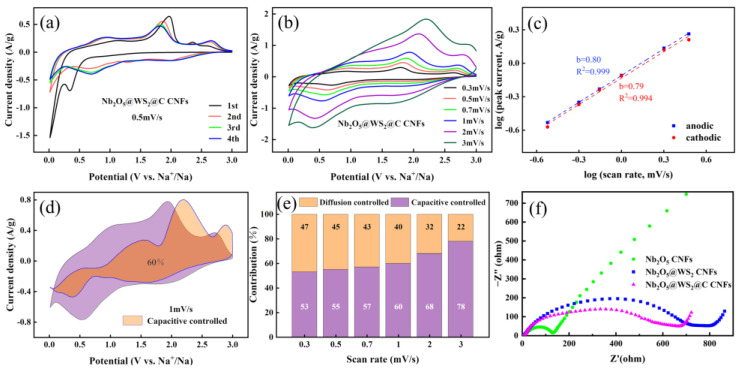
(**a**) The CV profiles of the initial four cycles for Nb_2_O_5_@WS_2_@C CNF electrode at a scan rate of 0.5 mV s^−1^. (**b**) The CV profiles of the Nb_2_O_5_@WS_2_@C CNF electrode at various scan rates from 0.3 to 3 mV s^−1^. (**c**) The relationship between peak currents and scan rates, (**d**) capacitive contribution curves at the scan rate of 1 mV s^−1^, and (**e**) normalized ratio of capacitive and diffusion at different scan rates, for Nb_2_O_5_@WS_2_@C CNF electrode. (**f**) EIS results of Nb_2_O_5_ CNFs, Nb_2_O_5_@WS_2_ CNFs, and Nb_2_O_5_@WS_2_@C CNFs.

Cyclic voltammetry (CV) tests were conducted to investigate the sodium storage mechanism during the charge and discharge processes. The CV profiles of the initial four cycles of different electrodes at a scan rate of 0.5 mV s^−1^ in the voltage range of 0.01 to 3.00 V vs. Na^+^/Na are shown in Figure 7a. Similar to other reports, Nb_2_O_5_ CNFs show no distinct oxidation and reduction peaks, making them a typical intercalation-based sodium storage material [18,36]. In contrast, the CNFs@WS_2_ electrode exhibits two reduction peaks at 0.85 and 0.38 V. In this case, the weak reduction potential at around 0.85 V is attributed to the intercalation of Na^+^ ions into WS_2_ (WS_2_ + xNa^+^ + xe^−^ → Na_x_WS_2_). The main peak at approximately 0.38 V is caused by the conversion reaction between WS_2_ and Na^+^ ions, leading to the intercalation of metallic W into the amorphous Na_2_S matrix and the formation of the SEI film (Na_x_WS_2_ + (4 − x)Na^+^ + (4 − x)e^−^ → 2Na_2_S + W). The oxidation peaks at 2.0, 2.3, and 2.6 V correspond to the process of sodium deiodination (Na_2_S → S + 2Na^+^ + 2e^−^; 2Na_2_S + W → WS_2_ + 4Na^+^ + 4e^−^). In the second to fourth cycles, reduction peaks at 0.7, 1.3, and 2.0 V and oxidation peaks at 1.0, 1.8, and 2.6 V were observed, indicating the presence of multi-step redox reactions. For the Nb_2_O_5_@WS_2_@C CNF electrode, apart from the first cycle corresponding to the formation of the SEI film, the curves of the second to fourth cycles essentially overlap, demonstrating excellent reversibility in Na^+^ storage. Additionally, the positions of the oxidation–reduction peaks closely align with those of the CNFs@WS_2_ electrode, indicating that the primary redox reactions are associated with WS_2_.

To further investigate the electrochemical behavior, we conducted CV tests on the Nb_2_O_5_@WS_2_@C CNF electrode at a scan rate within the range of 0.3 to 3 mV s^−1^. The relationship between current (i) and scan rate (v) is shown as follows:

i = av^b^ (1)

log(i) = blog(v) + log(a)(2)

Formula (2) is derived from Formula (1). The magnitude of the b value reflects the electrochemical behavior. When the b value approaches 0.5, it indicates diffusion-controlled behavior; when the b value approaches 1, it signifies that surface-controlled pseudocapacitive behavior predominates. As shown in Figure 7b, it is evident that the cathode and anode b values for the Nb_2_O_5_@WS_2_@C CNF electrode are 0.79 and 0.80, respectively. This suggests that the sodium-ion storage process is primarily governed by surface pseudocapacitive behavior. 

The following formula can be used to quantitatively analyze the capacity ratio of diffusion/capacitive-controlled processes:

i(V) = k_1_v + k_2_v^1/2^ (3)

where k_1_v and k_2_v^1/2^ represent the capacitive-controlled and diffusion-controlled processes, respectively. The values of k_1_ and k_2_ can be calculated by plotting i/v^1/2^ versus v^1/2^ at a fixed V-value with various scan rates. By calculation, at a scanning rate of 1 mV s^−1^, it is determined that the electrode’s capacitive control ratio is 60%. Moreover, with the scanning rate increasing from 0.3 to 3 mV s^−1^, the capacitive control ratio rises from 53% to 78%. The prominence of the pseudocapacitive process is due to the presence of more redox reaction sites on the surface of the anode material, which confers fast reaction kinetics and thus superior rate performance. Apparently, the sodium storage mechanism based on adsorption–intercalation–conversion in the Nb_2_O_5_@WS_2_@C CNF electrode favors a fast charge/discharge process on the surface of the active material. The electrochemical impedance spectra (EIS) testing of the Nb_2_O_5_ CNFs, Nb_2_O_5_@WS_2_ CNFs and the Nb_2_O_5_@WS_2_@C CNF electrode was conducted to further investigate the reaction kinetics. In the Nyquist plot, a typical semicircle and a straight line can be observed. The semicircle in the high-frequency range typically corresponds to the resistance of Na^+^ through the SEI film and charge transfer resistance, while the straight line in the low-frequency region is associated with diffusion impedance. The equivalent circuit diagram and corresponding values are shown in Table S2. Clearly, the semicircular region of the Nb_2_O_5_ CNF electrode has a smaller diameter, indicating lower resistance. With the introduction of WS_2_, the resistance of the entire electrode rapidly increases, corresponding to the relatively poor kinetics of WS_2_. Finally, the addition of soft carbon enhances the conductivity of the electrode, and the reduction in the semicircle diameter implies lower resistance and faster charge transfer kinetics.

Certainly, combining materials with different sodium storage mechanisms is advantageous for fully exploiting the strengths of each material. In this context, PAN-derived carbon serves as the organic framework, providing the foundation for a free-standing material. Nb_2_O_5_, being a typical insertion-based sodium storage material, exhibits a relatively low theoretical specific capacity. However, during the charge and discharge processes, it shows minimal volume expansion, demonstrating excellent rate capability and cycling stability. Additionally, the uniform dispersion of Nb_2_O_5_ particles contributes to good flexibility. On the other hand, WS_2_ belongs to conversion-based materials, possessing a higher theoretical specific capacity. Yet, limitations such as poor rate kinetics and substantial volume expansion restrict its applications. The mesh-structure carbon nanofibers provide a framework for the growth of WS_2_, which can effectively prevent the aggregation of WS_2_ during charge–discharge cycling, and most importantly, the addition of the carbon layer will not only increase the conductivity of the material but also inhibit the volume expansion during cycling. Furthermore, the carbon layer prevents direct contact between WS_2_ and the electrolyte, thereby inhibiting the dissolution of WS_2_.

## 4. Conclusions

In summary, we have successfully designed and fabricated a flexible sodium storage anode electrode based on the synergistic effects of multiple mechanisms. Initially, Nb_2_O_5_ was uniformly dispersed within PAN-derived carbon nanofibers through electrospinning and subsequent annealing, providing a free-standing framework and flexibility for the composite electrode. Subsequently, a hydrothermal process successfully synthesized WS_2_ and a carbon layer on the carbon nanofibers, ultimately resulting in the unique three-dimensional flexible Nb_2_O_5_@WS_2_@C CNF electrode. After testing, sodium storage materials with different sodium storage mechanisms in the composite electrode are able to leverage their respective advantages, significantly improving the material’s capacity density, rate performance, and cycling stability. Additionally, since the entire electrode is composed of active materials without the need for current collectors, binders, and conductive additives, it is expected to help improve the energy density of the entire battery.

## Data Availability

Data are contained within the article and Supplementary Materials.

## Supplementary Materials

The following supporting information can be downloaded at https://www.mdpi.com/article/10.3390/nano14070631/s1: Figure S1: Photograph of pure CNF films which were folded many times; Figure S2: TG curve of Nb_2_O_5_ CNFs; Figure S3: The GCD curve of Nb_2_O_5_ CNF, Nb_2_O_5_@WS_2_ CNF, Nb_2_O_5_@WS_2_@C CNF, and CNFs@WS_2_ electrodes at the current density of 0.5 A g^−1^; Figure S4: The CV profiles of initial four cycles for (a) Nb_2_O_5_ CNF, (b) Nb_2_O_5_@WS_2_ CNF, and (c) CNFs@WS_2_ electrodes at a scan rate of 0.5 mV s^−1^; Table S1: The results of elemental analysis; Table S2: The corresponding equivalent circuit and the calculated resistance values of the Nb_2_O_5_ CNFs, Nb_2_O_5_@WS_2_ CNFs, and Nb_2_O_5_@WS_2_@C CNFs.
