# Supplementary material for: Hybrid-Mechanism Synergistic Flexible Nb2O5@WS2@C Carbon Nanofiber Anode for Superior Sodium Storage
Source: Nanomaterials (Basel). 2024 Apr 5;14(7):631. doi: 10.3390/nano14070631 (PMC11013061; doi:10.3390/nano14070631)
Supplement: Supplementary file 1 [file nanomaterials-14-00631-s001.zip › nanomaterials-2931372-supplementary.pdf]

Supporting information

Hybrid-Mechanism Synergistic Flexible Nb_2O_5@WS_2@C Carbon Nanofiber Anode for Superior Sodium Storage

Yang Zhao [1,2], Ziwen Feng [1,2], Yipeng Tan [1,2], Qinglin Deng [1,2,*] and Lingmin Yao [1,2,3,*]

[1] School of Physics and Materials Science, Guangzhou University, Guangzhou 510006, China; 2112119105@e.gzhu.edu.cn (Y.Z.); 2112119037@e.gzhu.edu.cn (Z.F.); 2112119039@e.gzhu.edu.cn (Y.T.)
[2] Research Center for Advanced Information Materials (CAIM), Huangpu Research & Graduate School of Guangzhou University, Guangzhou 510555, China
[3] Joint Institute of Guangzhou University & Institute of Corrosion Science and Technology,
Guangzhou University, Guangzhou 510275, China
* Correspondence: qldeng@gzhu.edu.cn (Q.D.); lingminyao@gzhu.edu.cn (L.Y.)

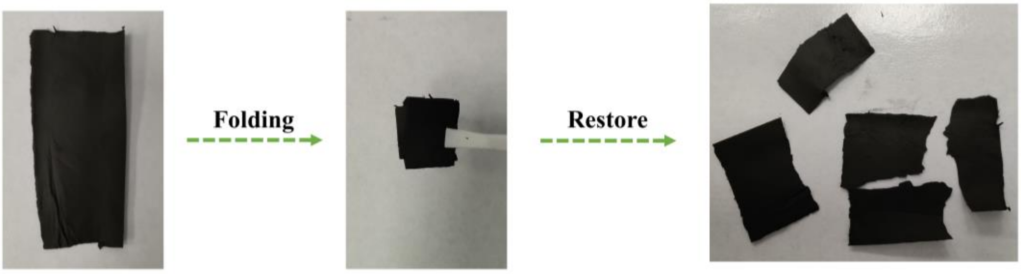

Figure S1. Photograph of pure CNFs films which were folded by many times.

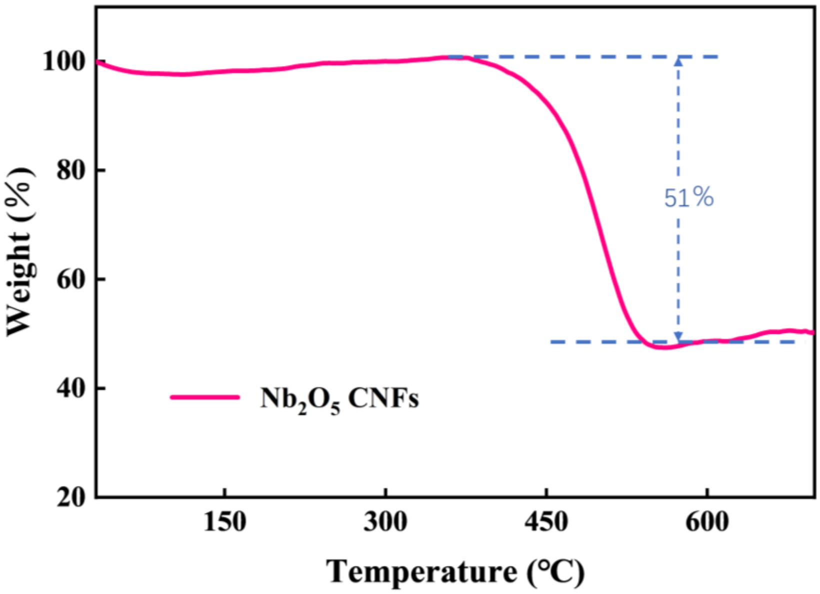

Figure S2. TG curve of Nb_2O_5 CNFs.

Table S1. The results of elemental analysis

Elemental	C (%)	S (%)	O (%)
**Percentage of quality**	16.8	18.2	4.1

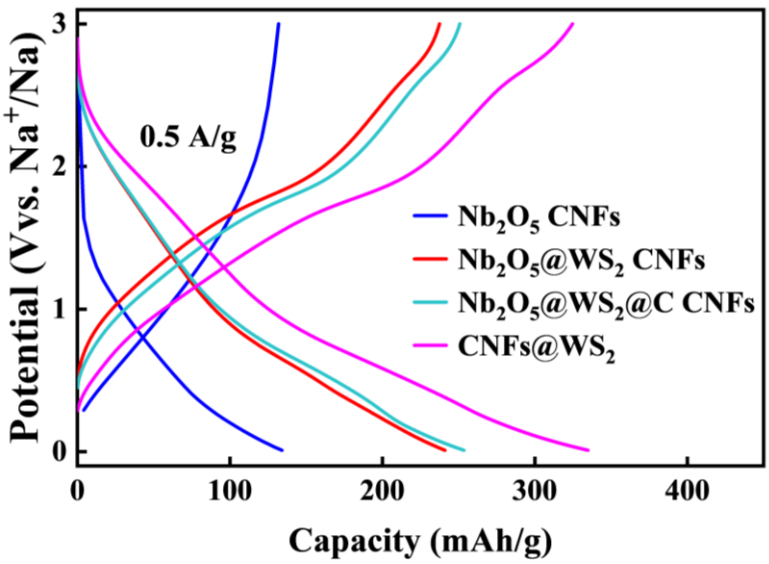

Figure S3. The GCD curve of Nb_2O_5 CNFs, Nb_2O_5@WS_2 CNFs, Nb_2O_5@WS_2@C CNFs and CNFs@WS_2 electrodes at the current density of 0.5 A g^{-1}.

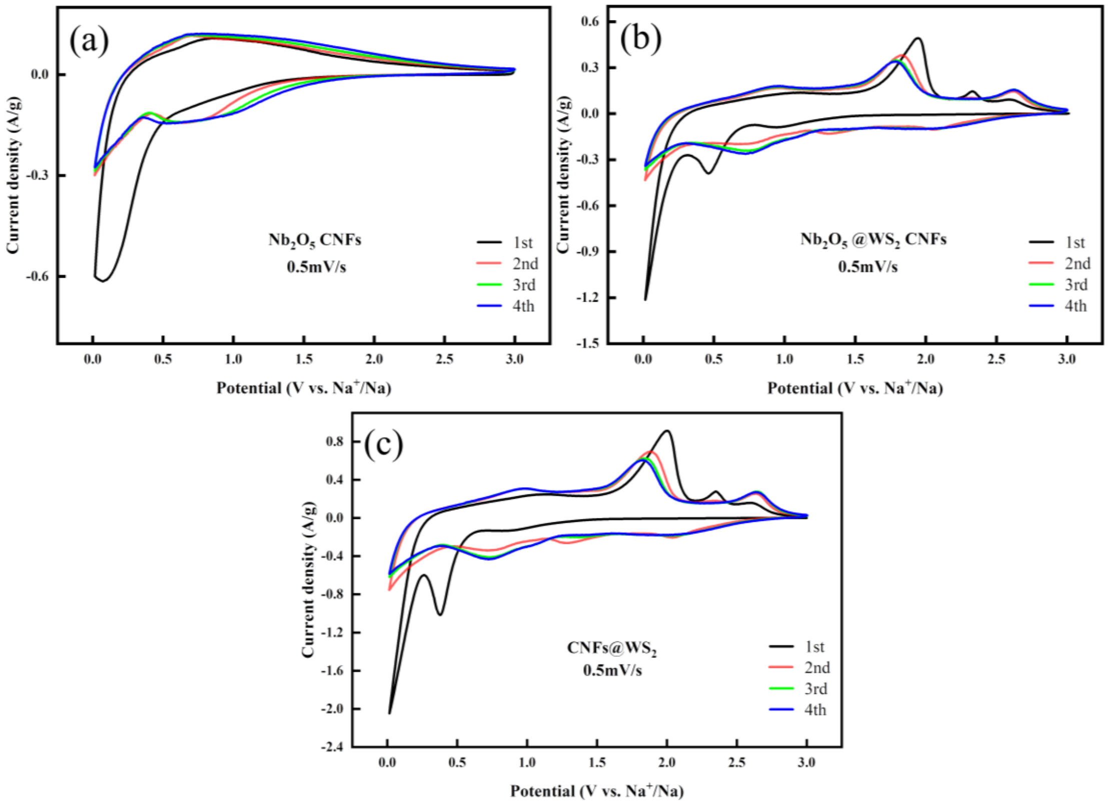

Figure S4. The CV profiles of initial four cycles for (a) Nb_2O_5 CNFs, (b) Nb_2O_5@WS_2 CNFs and (c) CNFs@WS_2 electrodes at a scan rate of 0.5 mV s^{-1}.

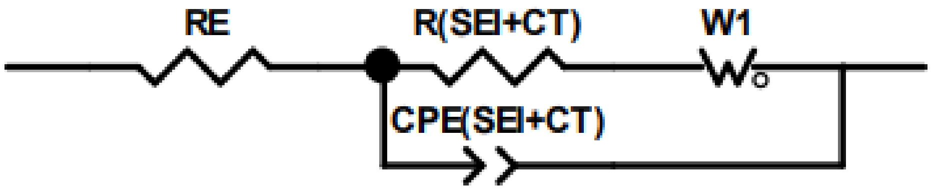

Table S2. The corresponding equivalent circuit and the calculated resistance values of the Nb_2O_5 CNFs, Nb_2O_5@WS_2 CNFs and the Nb_2O_5@WS_2@C CNFs.

**Electrodes**	**$R_{(SEI+CT)}$**
Nb_2O_5 CNFs	131.2
Nb_2O_5@WS_2 CNFs	729.4
Nb_2O_5@WS_2@C CNFs	613.4
